# Relationship Between Capillaroscopic Alterations and Bone Ultrasound Parameters in Patients with Raynaud Phenomenon

**DOI:** 10.2174/1874312900802010013

**Published:** 2008-02-28

**Authors:** Alfredo M Lurati

**Affiliations:** Rheumatology Unit Fornaroli Hospital, Magenta, Italy

**Keywords:** Raynaud phenomenon, QUS

## Abstract

The aim of this study was to evaluate phalangeal bone quantitative ultrasound (QUS) parameters in patients with Raynaud phenomenon (RP) and relate it with nailfold capillaroscopy findings. Patients referring to our Rheumatology Unit with RP were enrolled and studied for capillaroscopy alterations; bone quality profile was measured by QUS of the phalanxes**: **AD-SoS (Amplitude Dependent Speed of Sound) UBPI (Ultrasound Bone Profile Index), UBI (ultrasound Bone Index), Z score and T score were collected. One hundred thirty six females with RP had investigated for age, height, weight, Body Mass Index, previous diseases and therapies, menopausal age were enrolled. Nailfold capillaroscopy revealed minor alterations (borderline capillary dilatation, no capillary loss) in 36.8% (Group I), major alterations (capillaries definitely dilated, avascular areas, microbleeding) in 37.5% (Group II) and no significative alterations in 25.7% of patients (Group 0). A higher frequency of low QUS parameters in phalanxes was observed in group II when compared to group I or 0 (72.5% *vs* 54% *vs* 18%; p<0.01). With an ANOVA analysis we found a significant difference between the three groups in terms of Ad-SOS (Group II 1750±140; Group I 1890±132; Group 0 1990±167, p<0.001), UBPI (Group II 0.21±0.17; Group I 0.36±0.21; Group 0 0.51±0.24, p<0.001), UBI (Group II 1.2±0.43; Group I 1.4±0.32; Group 0 1.5±0.41, p<0.001), Z-scores (Group II -2.8±1.45; Group I -1.85±1.27; Group 0 -1.1±1.39, p<0.001) and T-scores (Group II -4.8±2.1; Group I -3.2±1.8; Group 0 -1.8±2.4, p<0.001). A standard linear regression analysis revealed an association between the capillaroscopy findings and QUS (R 0.47±0.8, p<0.01). In our study patients with capillaroscopy alterations showed reduced phalangeal quantitative ultrasound parameters, more markedly in patients with scleroderma pattern or other major capillaroscopy alterations, independently from confounding variables.

## INTRODUCTION

Raynaud’s phenomenon (RP) is a vasospastic disorder characterized by episodic color changes of blanching, cyanosis, and hyperemia in response to cold and/or emotional stress. RP may be a primary or a secondary process, primarily connective tissue diseases (CTDs). and scleroderma.

Nailfold videocapillaroscopy can describe and quantify the morphological characteristics of capillaries and distinguish different forms of connective tissue disease. Furthermore, low bone mass has been seen to occur in a significant percentage of patients with scleroderma or CTDs but few data are available up to date on the usefulness of quantitative ultrasound in the study of phalangeal bone quality and structure in primary and secondary RP. Aim of our study was to relate capillaroscopy alterations with bone quantitative ultrasound parameters (QUS) in patients with RP hypothizing that vascular alterations occurring in RP results in local lower bone quality and QUS values.

## MATERIALS AND METHODS

### Subjects

We studied females with RP, referring to our Rheumatologic division, at the Fornaroli Hospital of Magenta (Italy), from September 2006 to April 2007. A detailed history was taken of each patient, with particular reference to age, RP onset age, menopausal status, disease duration, current or previous treatments, and current or previous diseases; their height and weight were measured and related by the body mass index ratio (BMI). The following serological markers were determined: ESR, CRP, antinuclear antibodies, anticentromere antibodies, anti-extractable nuclear antigen, including anti-Scl70, Sm, RNP, SSB, SSA, and Jo1. Subjects were excluded if they had any history of a medical disorder known to affect bone metabolism or were on any medication that could affect bone mass, including use of corticosteroids or heparin, thyroid alterations, cancer, haemocromatosis, diabetes mellitus.

### Methods

For this study we defined Raynaud's phenomenon as episodic well-demarcated hands pallor and/or cyanosis in response to cold or emotional stress that was relieved by rewarming. We measured bone ultrasound parameters at phalanges: the amplitude-dependent speed of sound (AD-SoS), broadband ultrasound attenuation (BUA), UBPI (Ultrasound Bone Profile Index), UBI (ultrasound Bone Index), fast wave amplitude (FWA), which is the maximum amplitude of the fastest peak of the received US signal; Z score and T score values (referred to healthy Italian population) were also collected. Furthermore we performed a nailfold capillaroscopy on four fingers of both hands; all examinations were performed by the same experienced rheumatologist (MGM).

All patients were thereafter clinically evaluated by another experienced rheumatologist (AML) blind to the capillaroscopy results. The clinical diagnosis was used as gold standard.

### Statistical Analysis

All values were expressed as mean ± SD. An analysis of variance, simple (ANOVA) or with adjustment for some covariates (ANCOVA), was applied to compare the parameters measured between groups. Linear regression analysis and stepwise multiple regression analysis were used to assess the relationships between variables collected. A p value lower than 0.05 was considered statistically significant. All statistical analyses were carried out with SPSS software.

## RESULTS

**Fig. (1):**
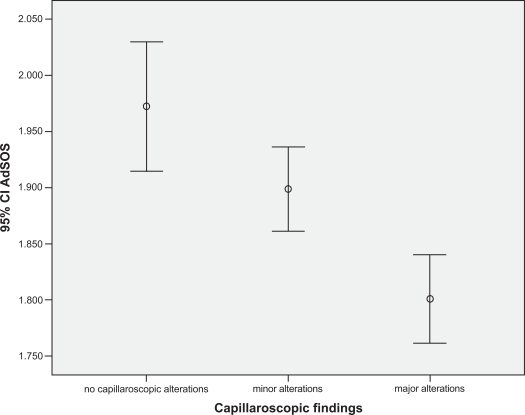
Ad-SOS mean values related to capillaroscopy pattern (p<0.001).

**Fig. (2):**
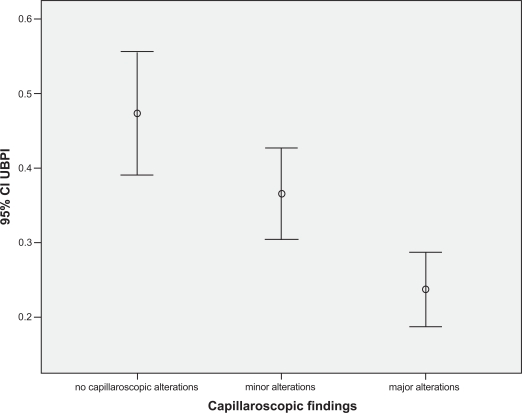
UBPI mean values related to capillaroscopy pattern (p<0.001).

**Fig. (3):**
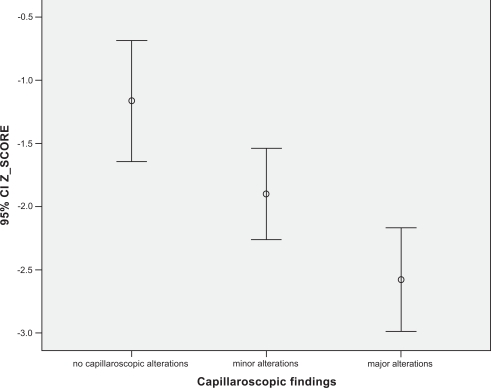
Z score mean values related to capillaroscopy pattern (p<0.001).

One hundred thirty six females with RP were enrolled. In whole population mean (±SD) age was 53.3 years (±14.1), mean BMI was 24.8(±7.8), mean menopausal age was 46.9 (±5.4). The mean QUS parameters values were: AdSOS 1881 (±159), FWA 28.7 (±9.8), UBI 1.32 (±0.38), UBPI 0.34 (±0.22). The mean Z score and T score were -1.98 and -3.4 respectively. Nailfold capillaroscopy revealed minor alterations (defined as: some tortuosity, borderline capillary dilatation, no capillary loss) in 36.8% (Group I), major alterations (defined as: capillaries definitely dilated, avascular areas, megacapillaries, microbleeding and capillary thrombosis, angiogenesis) in 37.5% (Group II) and no significant alterations in 25.7% of patients (Group 0). No differences were present in age, menopausal age, BMI between groups (p>0.2). In group II we found: Ad-SOS 1750±140, UBPI 0.21±0.17, UBI 12±0.43, Z-score -2.8±1.45 and T-score -4.8±2.1; with a standard ANOVA with post hoc test all these values were significantly lower than group I and group 0, also correcting for all confounding variables collected. (p<0.01) (Figs. **1-3**).

With a linear logistic test we found a Pearson R correlation index of: 0.37±0.7 for Z-score, 0.41±0.5 for UBPI and 0.47±0.8 for AdSOS (p<0.001).

The conclusive clinical evaluation of the whole population revealed that the 88% of group II patients, 15% of group I patients and 1.7% of group 0 were affected by a major CTD including SSc (p<0.001). In all other cases a diagnosis of primary Raynaud was made.

## DISCUSSION

Raynaud’s phenomenon (RP) is a common clinical condition in rheumatology, with microvascular involvement as key feature. LeRoy and Medsger suggested criteria for primary RP: symmetric attacks, the absence of tissue necrosis, ulceration or gangrene, the absence of a secondary cause, negative antinuclear antibodies, normal nailfold capillaroscopy and a normal erythrocyte sedimentation rate. Secondary RP is characterized by an age of onset of more than 30 years, painful and asymmetric attacks, ischemic skin lesions, positive autoautoantibodies, capillaroscopy major abnormalities and/or clinical features suggestive of CTDs. Among the CTDs, scleroderma has the highest frequency of RP. Peripheral microangiopathy may be recognized by nailfold capillaroscopy, a non-invasive and safe technique that is reported to have both diagnostic and prognostic value. The peripheral microvascular damage in scleroderma is characterized by increasing structural alterations of the capillaries (giant capillaries and microhaemorrhages) with a progressive decrease in their density (scleroderma pattern). Generalized radiological osteopenia has been seen to occur in a significant percentage of patients with connective diffuse diseases and scleroderma. Bone mineral content was found to be reduced at the radius, lumbar spine, and the total body. No data are available up to date on QUS evaluation of phalangeal bone in patients with primary or secondary RP [7-9]. Recently there has been a growing scientific and clinical interest in QUS as an alternative method to dual-energy X-ray absorptiometry (DXA) for assessing skeletal status. QUS is thought to reflect not only bone density, but also some structural properties of bone, such as elasticity and trabecular stiffness and connectivity, which could be closely connected to bone strength [10, 11]. Up to date no data are present in literature about bone quality parameters or bone mass indices of hand fingers phalanxes, that are primarily affected during RP. In our study we used QUS instead DEXA for studying not only bone density but primarily bone quality. Our results seems confirming literature, showing that patients with major capillaroscopy alterations are affected more likely by CTDs and are characterized by generalized osteopenia or osteoporosis. Finally, also the bone quality indices seems to be altered, suggesting a bone sufferance caused by local microvascular damages.

## CONCLUSION

Data collected in our study suggest that RP, primarily in patients with scleroderma other CTDs, modify phalangeal bone mass, bone density, and bone quality with a good correlation between nailfold patterns and QUS, independently from confounding variables.
